# Comparison of Robotic Posterior Retroperitoneal Adrenalectomy over Laparoscopic Posterior Retroperitoneal Adrenalectomy: A Single Tertiary Center Experience

**DOI:** 10.1155/2019/9012910

**Published:** 2019-12-01

**Authors:** Won Woong Kim, Yu-mi Lee, Ki-Wook Chung, Suck Joon Hong, Tae-Yon Sung

**Affiliations:** Department of Surgery, Asan Medical Center, University of Ulsan College of Medicine, Seoul 05505, Republic of Korea

## Abstract

**Background:**

The aim of this study is to compare the clinical outcomes of laparoscopic posterior retroperitoneal adrenalectomy (LPRA) and robotic posterior retroperitoneal adrenalectomy (RPRA) and determine the differences that could affect the outcomes.

**Methods:**

We retrospectively analyzed 230 adrenalectomy cases from 2014 to 2017. There were 169 LPRA and 61 RPRA cases, and their clinicopathological features and surgical outcomes were compared.

**Results:**

In LPRA, there was a positive relationship between operation time and male gender, early period of experience, adrenal tumor size, and pheochromocytoma. In RPRA, adrenal tumor size and pheochromocytoma were the factors affecting the operation time. When the adrenal tumor size was ≤5.5 cm, the operation time of LPRA was shorter than that of RPRA (*p*=0.001). When the tumor size was >5.5 cm, there was no significant difference in the operation times of LPRA and RPRA (*p*=0.102).

**Conclusions:**

RPRA is a feasible and technically safe approach for benign adrenal diseases. The use of RPRA could benefit patients and provide comfort by overcoming the factors contributing to a longer operation time in the laparoscopic technique, such as male gender and high BMI.

## 1. Introduction

The laparoscopic transperitoneal adrenalectomy (LTA) approach was first introduced in 1992 [1]. Subsequently, LTA has been found to have several benefits compared with open adrenalectomy, such as reduced postoperative pain, less blood loss, decreased wound complication rate, reduced length of hospital stay, and superior cosmesis [2–5]. Alternative approaches, such as lateral retroperitoneal or posterior retroperitoneal adrenalectomy (PRA), have been developed to eliminate the need for mobilization of adjacent structures and to reduce the risk of associated complications [6–8]. Recently, laparoscopic PRA (LPRA) has demonstrated excellent surgical outcomes compared with LTA despite disadvantages such as a small working space and cardiovascular compromise due to higher insufflation pressures in PRA [9–20]. In cases of large tumors, surgeons prefer LTA over LPRA, with the presumption of a limited working space [21]. Nevertheless, LPRA has the advantages of a significantly shorter operation time, less postoperative pain, and less estimated blood loss (EBL). It is also recommended in patients with abdominal adhesions and those with bilateral tumors [22, 23]. In addition, the robotic PRA (RPRA) approach has been proposed to achieve better outcomes in certain cases, especially with the limited working space in the posterior retroperitoneal approach [12, 24, 25].

In this study, we aimed to compare the clinical outcomes of LPRA and RPRA and to determine the differences that could affect the outcomes. In addition, we analyzed the factors that could be associated with increased operation time for each adrenalectomy approach.

## 2. Materials and Methods

### 2.1. Patients

**Figure 1:**
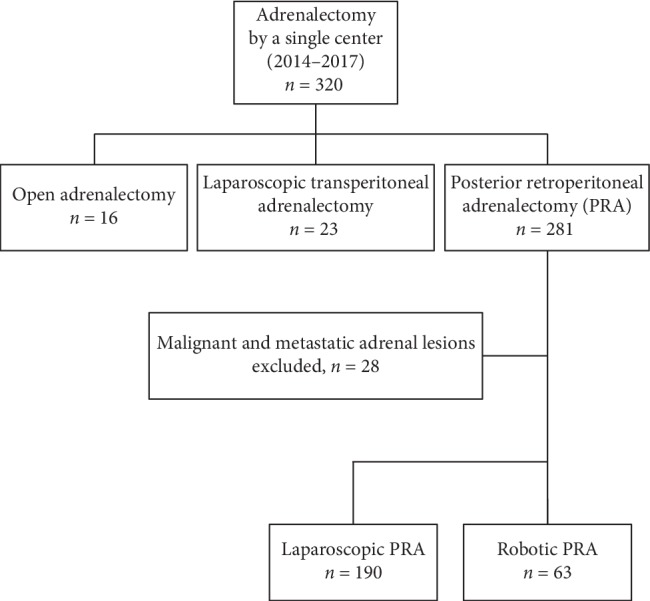
Selection of the study population.

We retrospectively analyzed 320 adrenalectomy cases by a single experienced surgeon at Asan Medical Center from January 2014 to December 2017. There were 16 cases of open adrenalectomy, 23 of LTA, and 281 of PRA. For the open adrenalectomy and LTA cases, patients with malignant tumors, such as adrenocortical carcinomas and malignant pheochromocytomas, and metastatic adrenal lesions from other primary carcinomas who underwent PRA were excluded (*n* = 28). Eleven bilateral adrenal disease cases and combined operation cases were also excluded. After exclusions, 230 cases (169 LPRA and 61 RPRA cases) were evaluated (Figure 1). All of the unilateral adrenalectomy cases were performed with complete resection of the involved adrenal gland. The present study protocol was reviewed and approved by the Institutional Review Board of Asan Medical Center, and informed consent was waived as this was a retrospective study. All methods were performed in accordance with the relevant guidelines and regulations.

Various clinicopathological features, including the age at operation, gender, adrenal tumor size, height, weight, body mass index (BMI), length of hospital stay, type of disease, adrenal tumor site, estimated blood loss (EBL), and mean operation time, were assessed. For learning curve analysis, we defined the early and late periods of experience as the first and second years, respectively, of performing each approach (LPRA and RPRA).

### 2.2. Surgical Procedure

The selection of the conventional open or laparoscopic approach depended on a decision-making process based on various published studies and the surgeon's preference which was based on each individual patient's characteristics or the anatomic and pathological features of the adrenal gland tumors. The selection of LPRA or RPRA depended on the patient's preference based on the individual patient's characteristics and personal insurance coverage. LPRA was readily performed after 2014 by the surgeon, who had years of experience with LTA, and RPRA was performed after the start of 2016 when the surgeon's PRA experience was past the learning curve of performing more than 50 LPRA operations. For the LPRA and RPRA approaches, three port-site incisions were made as previously described [26]. In this study, the operation time included surgical draping, preparing the operative field (with patient cart docking for RPRA), the main tumor resection procedure, irrigation and drainage, extraction of the specimen, and wound closure. Insertion of the drainage tube was not considered in most cases, unless the irrigation and drainage amount was more than 500 ml. For RPRA, we used an operating room with a robotic system always ready for use, which required no additional time for preparation.

### 2.3. Statistical Analysis

Student's *t*-test was used to assess between-group differences with respect to continuous variables. *χ*^2^ test or Fisher's exact test was used to compare categorical variables. Continuous variables are presented as the mean ± standard deviations with ranges, and categorical variables are presented as percentages and absolute numbers. Multivariate linear regression modeling analysis was performed to identify factors that could increase the operation time. Scatter plots with Pearson's correlation coefficients were used to show the relationship between adrenal tumor size and operation time. Beta coefficients with 95% confidence intervals (CIs) were calculated. A *p* value of <0.05 was considered statistically significant. Analyses were performed using SPSS version 20.0 for Windows (IBM Corp., Armonk, NY, USA).

## 3. Results

The clinicopathological characteristics of the 230 patients are shown in Table 1. The mean age of the patients was 49 years, and there were 85 males and 145 females. The mean size of the adrenal tumor was 3.5 cm, and the mean BMI was 24.8. The mean length of hospital stay was 4 days. Pheochromocytoma was the most frequently operated disease (78, 33.9%), followed by Cushing's syndrome (69, 30.0%) and primary aldosteronism (51, 22.2%). The mean operation time was 118 min. Among the 230 patients, 169 patients underwent LPRA (73.5%) and 61 patients underwent RPRA (26.5%). There were no differences in the gender, adrenal tumor size, height, weight, BMI, length of hospital stay, type of disease, or adrenal tumor site of the two groups. The mean operation time of the LPRA group was significantly shorter than that of the RPRA group (117 vs. 142 min, *p*=0.006) (Table 1). In addition, for operation time evaluation, 10 bilateral cases in the LPRA group and 1 bilateral case in the RPRA group were excluded to analyze the operation time in a uniform range. Mean EBL was <100 cc in both approaches, showing no considerable difference, and there was no conversion to open technique in our study. There were no morbidity- or mortality-related complications in this study population.

As there was a significant difference in the operation times, factors that could affect the length of operation were evaluated. Univariate and multivariate analysis revealed that male gender, BMI, adrenal tumor size, type of disease (pheochromocytoma), and RPRA were related to operation time in the PRA group (Table 2). After classifying the cases according to the type of PRA, multivariate analysis revealed that male gender, early period of experience, adrenal tumor size, BMI, and type of disease (pheochromocytoma) were significantly related to a longer operation time in the LPRA group. However, in the RPRA group, the adrenal tumor size and type of disease (pheochromocytoma) were significantly related to the length of operation.

**Figure 2:**
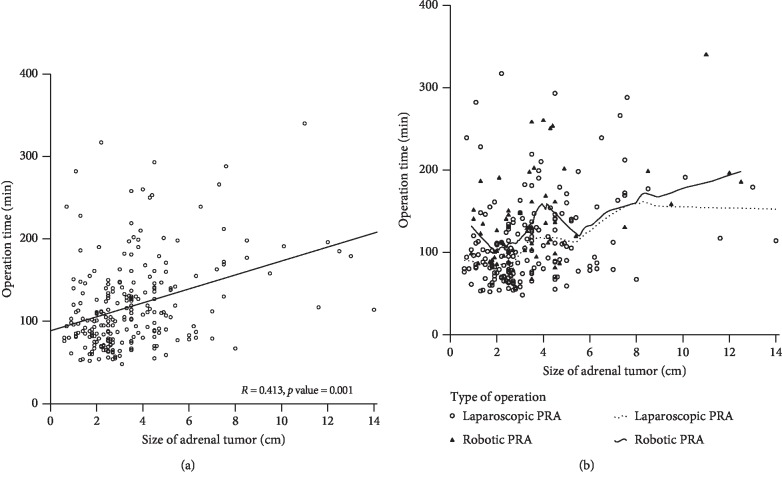
Scatter plots of the adrenal tumor size and operation time. (a) Correlation between operation time and posterior retroperitoneal adrenalectomy (PRA) cases. (b) Comparison of laparoscopic PRA and robotic PRA cases.

Analysis of the correlation between the adrenal tumor size and operation time revealed a positive correlation (*R* = 0.413, *p* < 0.001; Figure 2(a)). When the adrenal tumor size was ≤5.5 cm, the operation time of LPRA was shorter than that of RPRA, and when the size was >5.5 cm, a parallel line was observed up to a certain point (Figure 2(b)).

**Figure 3:**
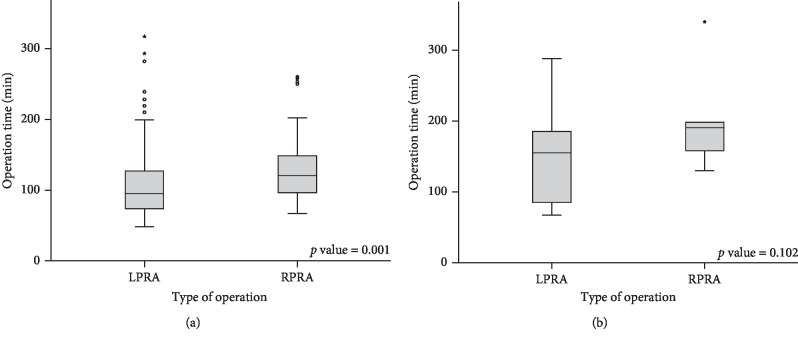
Box plots of the operation time in laparoscopic posterior retroperitoneal adrenalectomy (LPRA) and robotic posterior retroperitoneal adrenalectomy (RPRA) cases according to the adrenal tumor size. (a) Tumor size ≤ 5.5 cm. (b) Tumor size > 5.5 cm.

In cases with ≤5.5 cm adrenal tumors, the operation times of LPRA and RPRA were 105 min and 131 min, respectively (*p*=0.001, Figure 3(a)). On the other hand, in cases with >5.5 cm tumors, there was no difference in the operation times of LPRA and RPRA (155 vs. 190 min, *p*=0.102; Figure 3(b)). Among tumors larger than 5.5 cm, the mean sizes of the adrenal tumor were 7.2 cm and 10.3 cm in the LPRA and RPRA groups, respectively (*p*=0.02). However, box plots revealed a wider range of operation times in the LPRA group than in the RPRA group (Figure 3).

## 4. Discussion

Laparoscopic adrenalectomy (LA) has become more acceptable among surgeons, and it is considered as the gold standard technique for removing certain adrenal masses [27–30]. In this study, we aimed to compare the clinical outcomes of LPRA and RPRA and determine the differences that could affect the outcomes. In addition, we hypothesized that certain factors may be associated with increased operation time.

The advantage of LA is well described in various studies [27, 28, 31]. However, the history of robotic adrenalectomy (RA) is relatively short, and it is performed only in certain institutions. The safety of RA has been investigated in previous studies, and the benefits of RA compared with LA are still being debated [12, 32]. Recent studies have reported that there are no differences between LPRA and RPRA in EBL, postoperative pain, complication, or conversion rate [13, 23, 33]. In addition, a shorter hospital stay has been reported for RPRA; however, the cost is higher. In this study, there were no differences in the age, gender, adrenal tumor size, height, weight, BMI, type of disease, or adrenal tumor site between the LPRA and RPRA groups. In addition, the length of hospital stay was not different between the LPRA and RPRA groups. Cost is a significant concern when performing the robotic procedure in Korea. Robotic surgery has been reported to be 1.2–3.2 times more costly than laparoscopy [20]. In Korea, the cost of RA is 3 times more expensive than LA as reported previously. Private insurance policies are rather complicated in Korea; however, if patients have the right insurance, they would pay an equal amount for either the RA or LA approach. As the patients' choice of an approach was based on their personal insurance coverage, cost should not be considered as a factor affecting outcome. However, the impact on medical insurance resources and infrastructure is considerable and should be considered with the technical feasibility and outcomes. We should consider which patients are appropriate for robotic approach considering the economic and social cost barriers.

In the previous studies, the operation time of RA was significantly longer compared with that of LA [13, 15]. Although an experienced surgeon performed RPRA, the extra time required to dock the robot increased the operation time [12, 34]. Similarly, in our study, the operation time of RPRA was longer than that of LPRA. In RPRA, an additional 25 min was required as the operation time included the preparation of the operative field with patient cart docking; this could have affected the operation time of RPRA. Furthermore, the additional time required may be attributed to overcoming the anatomical barrier and preparing the working space with the help of robotic-assisted technologies such as the magnification of camera views, instrument articulation, and rotation motion.

As there was a difference in operation time between the two groups, we analyzed the factors that could affect the length of operation in the LPRA and RPRA groups by multiple linear regression analysis. In the LPRA group, male gender, early period of experience, adrenal tumor size, obesity, and type of disease (pheochromocytoma) were associated with increased operation time. However, in the RPRA group, adrenal tumor size and type of disease (pheochromocytoma) were the factors related to the length of operation. Therefore, surgeons can perform RPRA with ease regardless of factors such as male gender and obesity. We could explain the reason of shorter operation time in certain situations that the robotic system has a wider range of wrist-part angulation and movement within the limited small space, which is an issue in laparoscopic procedures. This angulation movement made the surgeon more comfortable to manage the fats around the perirenal areas in this study. The operation time was increased with every centimeter increase in the adrenal tumor size, which increased the operation time by around 7.2 min (beta coefficient 7.2, *p* < 0.001; Table 2).

Various studies have demonstrated that LA for tumors larger than 5–8 cm is feasible and safe when performed by an experienced surgeon. Although the operation time of RA would be longer than that of LA, some studies have proposed that the use of a robotic approach could shorten the operation time for adrenal tumors larger than 5 cm [24, 35]. In our study, the operation time of RPRA was longer than that of LPRA in cases with ≤5.5 cm tumors. However, the operation time was similar for both groups, without large differences in cases with >5.5 cm tumors. In addition, although the mean adrenal tumor size (>5.5 cm tumors) in the RPRA group was much larger than that in the LPRA group (10.3 cm and 7.2 cm, respectively), the operation time was not significantly different between the two groups (Figure 3(b)).

A limitation of this study was that the number of patients was small in the RPRA group compared with the LPRA group. Furthermore, this was a retrospective study with a selection bias because each approach was chosen based on the patient's individual characteristics and personal insurance coverage. Most of the cases included in this study had small tumor size and were benign. This could have acted as an additional bias for selecting PRA. However, the distribution of the type of disease and age, gender, height, weight, BMI, size of adrenal tumor, and site of adrenal tumor was not different between the two groups. In this study, we could not show the net benefit of the robotic system compared to the laparoscopic system. In addition, all surgery was performed by a single surgeon, and this could affect the representativeness of the technique as a limitation. However, we expect that the robotic system could have a role of conservation surgery since it has a wider range of angulation and movement.

## 5. Conclusion

In summary, RPRA may be a more feasible and safe approach compared with the known LPRA approach for benign adrenal diseases. In addition, the selective use of a robotic system with additional angulation movement may help surgeons to overcome the factors related to longer operation time in the laparoscopic technique, such as male gender and high BMI. However, the use of RPRA should be considered both in terms of cost-effectiveness and technical feasibility.

## Figures and Tables

**Table 1:** Clinicopathological characteristics of patients who underwent laparoscopic and robotic posterior retroperitoneal adrenalectomy.

Characteristics	Total (*n* = 230)	Laparoscopic PRA (*n* = 169)	Robotic PRA (*n* = 61)	*p* value
Age (years)	49.1 ± 13.0	50.1 ± 13.4	46.5 ± 11.6	0.059
Gender				0.242
Male	85 (37.0%)	66 (39.1%)	19 (31.1%)	
Female	145 (63.0%)	103 (60.9%)	42 (68.9%)	
Size of adrenal tumor (cm)	3.5 ± 2.2 (0.6, 14)	3.4 ± 2.2 (0.6, 14)	3.7 ± 2.5 (0.9, 12.5)	0.483
Height (cm)	163 ± 8.4	163.0 ± 8.7	163.3 ± 7.3	0.824
Weight (kg)	66.0 ± 11.9	65.9 ± 12.1	66.2 ± 11.3	0.849
BMI	24.8 ± 3.8	24.8 ± 3.9	24.8 ± 3.5	0.974
Length of hospital stay (days)	4.2 ± 2.6	4.2 ± 2.8	4.0 ± 1.8	0.626
Cushing's syndrome	5.4 ± 3.0	5.5 ± 3.4	5.2 ± 2.1	
Pheochromocytoma	4.0 ± 3.1	4.2 ± 3.6	3.5 ± 1.4	
Primary aldosteronism	3.3 ± 0.7	3.4 ± 0.7	3.0 ± 0.7	
Type of disease				0.309
Pheochromocytoma	78 (33.9%)	54 (32.0%)	24 (39.3%)	
Cushing's syndrome	69 (30.0%)	47 (27.8%)	22 (36.1%)	
Primary aldosteronism	51 (22.2%)	42 (24.9%)	9 (14.8%)	
Other benign diseases	32 (13.9%)	26 (15.3%)	6 (9.8%)	
Site of adrenal tumor				0.233
Right	114 (49.6%)	88 (52.1%)	26 (42.6%)	
Left	116 (50.4%)	81 (47.9%)	35 (57.4%)	
EBL				
Less than 100 cc	230	169	61	1.00
Mean operation time (range, min)	118 ± 53.2 (48–340)	110 ± 50.9 (48–317)	138 ± 54.5 (67–340)	0.001
Type of disease				
Pheochromocytoma	136 ± 56.1	126 ± 53.4	161 ± 55.2	0.009
Cushing's syndrome	100 ± 38.1	90 ± 38.3	119 ± 30.0	0.003
Primary aldosteronism	100 ± 45.2	99 ± 48.3	105 ± 28.0	0.713
Site of adrenal tumor				
Right	120 ± 55.4	113 ± 53.9	144 ± 55.0	0.014
Left	115 ± 51.1	106 ± 47.4	134 ± 54.7	0.006

PRA: posterior retroperitoneal adrenalectomy; BMI: body mass index.

**Table 2:** Univariate and multivariate analysis of factors related to the operation time.

Variables	Univariate analysis total (*n* = 230)		Multivariable analysis total (*n* = 230)		Laparoscopic PRA (*n* = 169)		Robotic PRA (*n* = 61)	
Related factors	Beta coefficient	*p* value	Beta coefficient	*p* value	Beta coefficient	*p* value	Beta coefficient	*p* value
Gender (male vs. female)	14.7	0.037	14.3	0.028	21.5	0.003	−12.2	0.363
Early period vs. late period (years)	6.3	0.118	8.9	0.010	11.1	0.004	−11.6	0.349
BMI								
>27 vs. ≤27	19.0	0.015	26.0	0.001	21.0	0.006	21.5	0.141
Size of adrenal tumor (cm)	12.2	0.001	7.2	0.001	5.6	0.001	9.7	0.001
Pheochromocytoma	29.0	0.001	19.5	0.003	16.8	0.03	31.3	0.019
Site (left vs. right)	3.8	0.580	7.6	0.216	4.6	0.514	−7.6	0.432
Robotic PRA	26.5	0.001	32.4	0.001				

PRA: posterior retroperitoneal adrenalectomy; BMI: body mass index.

## Data Availability

The datasets generated during and/or analyzed during the current study are available from the corresponding author on reasonable request.
